# Barium nanoparticles enhance efficacy of external beam radiation therapy in a preclinical basal-like mammary cancer mouse model

**DOI:** 10.1038/s41598-025-02560-4

**Published:** 2025-05-30

**Authors:** Jonas Albers, Andrea Markus, Angelika Svetlove, Alexander Kraupner, Andreas Briel, Frauke Alves, Christian Dullin

**Affiliations:** 1 Institute for Clinical and Interventional Radiology, University Medical Center Goettingen, Göttingen, Germany; 2 Translational Molecular Imaging, Max-Plank-Institute for Multidisciplinary Sciences, Göttingen, Germany; 3 NanoPet Pharma GmbH, Berlin, Germany; 4 Department for Haematology and Medical Oncology, University Medical Center Goettingen, Göttingen, Germany; 5 Institute for Diagnostic and Interventional Radiology, University Hospital Heidelberg, Heidelberg, Germany; 6 Italian synchrotron “Elettra”, Trieste, Italy; 7 Present Address: Hamburg Unit, c/o DESY, European Molecular Biology Laboratory, Notkestraße 85, Hamburg, Germany

**Keywords:** Radiation therapy, Contrast enhancer, Nanoparticle, Drug development, Medical research, Preclinical research

## Abstract

External beam radiation therapy (RT) using high energy x-rays is a commonly applied cancer treatment. A major advantage of RT is its unspecific nature which allows using RT in many cancer entities. RT however causes the well-known side effects on healthy tissue in the irradiated areas. Therefore, enhancing the efficacy of RT at the tumor site while simultaneously lowering the overall radiation dose has been a long term goal. Heavy metal-based contrast agents such as gold, hafnium, gadolinium and iodine have already been proposed as radio-enhancers and are partially in clinical trials. Here we present barium sulphate (BaSO_4_) nanoparticles as novel radio-enhancer for RT validated in a syngenic mouse breast cancer model. We demonstrate that these particles in combination with low energy RT significantly reduced tumor growth when compared to untreated controls and tumors that received RT only. Despite the fact that the absorption probability decreases with increasing photon energy, we see a stronger anti-tumoral effect at energies around 90 keV which would allow a translation of this approach into a clinical RT setting. Due to the strong contrast of barium in computed tomography such (BaSO_4_) nanoparticles could be used for both, better tumor delineation as well as for enhancing RT response.

## Introduction

Radiation therapy (RT) using high energy x-rays is one of the most commonly applied anti-cancer therapies^1^. RT exploits the fact that the highly proliferating tumor cells show impaired repair mechanisms against radiation-induced cell stress^2^. RT is therefore an unspecific therapy that can be applied in virtually any tumor entity. However, healthy cells in the irradiated area also receive radiation damage causing well-known side-effects^3^. In order to circumvent this aspect, many modifications of RT have been proposed, including the use of radio-enhancers - materials that increase the dose deposition within the tumor or initiate additional damaging effects^4^. Such materials are typically based on heavy ions like iodine, gadolinium, gold or hafnium^5^. However, even these elements with higher atomic numbers are virtually transparent at photon energies above 100 keV, which is the energy typically employed for RT to reduce the absorption of healthy tissue above the tumor. Thus radio-enhancers have been tested in combination with low energy RT (LE-RT) using photon energies in the range of 50 keV^6^. A successful approach of LE-RT in combination with radio-enhancers could in theory be performed with standard computed tomography (CT) systems, which would allow staging and therapy of the tumor in the same device and in one session. The mode of action of classical metal-based radio-enhancers is electron release by the photo-electric effect from the K, L or M shell^5^. The effect of those primary photo-electrons is limited by their average traveling range within the tumor tissue, which depends on their kinetic energy^7^. Thus, despite the fact that the photo-electron release of k-shell is highest at the k-edge of the material, a higher irradiation photon energy should be chosen to increase the kinetic energy of the resulting photo-electrons. The latter effect would also allow to shift the irradiation towards clinically relevant higher energies, which are used to reduce the dose deposition within superficial healthy tissue^5^. Clinical trials currently focus on Hafnium as for instance reported by Koshy et al.^8^ for novel nanoscale metal-organic frameworks (MOFS) or by Fuentes et al.^9^ for a phase 1 study in pancreatic ductal adenocarcinoma. Gold based radio-enhancers have already been studied in the past as for instance reported by Libutti et al.^10^ in combination with a recombinant tumor necrosis factor (rhTNF) antibody. In addition, also gadolinium is proposed as radio-enhancer^11^. Gadolinium is commonly applied as contrast agent for magnetic resonance imaging (MRI) which would allow to combine improved lesion detection with enhanced therapy outcome.

Here we propose to use Barium as a radio-enhancer, which with its comparable low k-edge energy of 37.4 keV could already show enhancing effects at photon-energies used for x-ray diagnosis such as computed tomography. Moreover, barium is well established as contrast agent for oral application in patients^12^ and as nano-particles for in-vivo small animal computed tomography studies^13^. Thus barium based radio-enhancers would potentially allow to enhance tumor delineation by computed tomography and enhanced treatment response with the same compound and therefore in a narrow time frame. A successful implementation of such an approach would require answering three main questions: (i) do the particles show toxic side effects and where do they end up after the therapy, (ii) can the particles be enriched at the tumor site and (iii) does the additional effect of the particles counter balance the need of using low energy photons?

Here we present the capacity of barium nanoparticles as radio-enhancers in a breast cancer mouse model^14^ in combination with LE-RT using preclinical in-vivo micro computed tomography (µCT) for enhancing the anti-tumoral effects in comparison to classical RT. We apply the novel concept to a murine breast cancer mouse model. Basal breast cancer (BLBC) and triple-negative breast cancer (TNBC), two largely overlapping BC subtypes, lack expression of currently druggable molecular targets. Since the therapeutic options for treating these malignant diseases are minimal and are usually limited to conventional chemotherapy and radiotherapy the development of efficient treatments for this specific disease subtype is of high medical need. Here we used murine H8N8 mammary carcinoma cells that were isolated from an endogenously induced tumor of the bitransgenic WAP-T-NP8xWAP-mutp53-H8 mouse line and thus exhibit characteristics of the difficult-to-treat BLBC/TNBC^15,16^. Upon orthotopic transplantation in syngeneic animals, this cell line generates poorly differentiated basal-like breast tumors harboring a high degree of epithelial-mesenchymal plasticity. This immunocompetent BLBC mouse model has been used for several in vivo studies to analyse the progression of BLBC and to preclinically validate novel therapies and chemoresistance.^15,17–19^.

## Results

### Dose measurements and characteristics of the irradiation devices

**Fig. 1:**
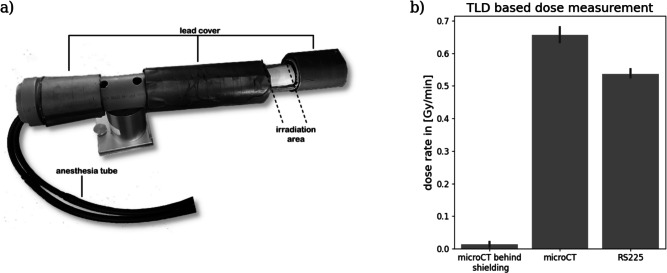
Irradiation strategy. (**a**) shows the custom made mouse holder for irradiation in the CT. Two circular lead covers (5 mm thickness) can be adjusted to limit the irradiation area to the size and position of the tumor. (**b**) shows the dose rates measured by the TLDs (embedded in dead mice in case of the CT). Only minimal dose deposition was measured behind the shielding. In the CT we reached a dose rate of approx. 0.65 Gy/min and in the RS225 of 0.55 Gy/min.

In our experiments, an in-vivo µCT and a cabinet irradiator (RS225) were used for irradiation at average beam energies of approx. 40 keV and 90 keV, respectively. Since the use of classical Geiger-Muller tube-based dosimeters would not have led to comparable results due to the different designs and photon energy ranges of the two different devices, we used thermoluminescence dosimeters (TLDs). TLD’s are small and can easily be inserted into any system. In the µCT we combined the dose measurements with the test of our customized mouse holder (Fig. 1a). We implanted the TLDs in 4 dead mice. Figure 1b shows that locations behind the lead shielding of the mouse holder barely received any dose deposition. In contrast, in the “irradiated area” a dose rate of approx. 0.65 Gy/min was measured. In case of the RS225 device the TLDs were positioned on the tray used for both mouse and cell irradiation. We measured a dose rate of approx. 1.1 Gy/min for the cell and 0.55 Gy/min for the in-vivo experiments. Despite the advantages of TLDs, the major limitation is the retrospective read-out. We thus only realized at the end of the in-vivo experiment, that only 50% of the intended dose rate had been delivered due to a malfunction in the device. Thus, the in-vivo experiments were performed with different total dose rates in µCT and RS225.

As indicated by Yarnold et al.^20^ a typical fractionated treatment of breast cancer is composed by 20 daily sessions with 2.0 Gy each. Thus, in our case mice would need to be irradiated for 3 mins in the CT and for 3.6 mins in the RS225 irradiation device. Since the CT acquisition protocols were fixed we used two 2 min acquisitions which resulted in a daily dose of 2.6 Gy. Tumor bearing mice were irradiated for 10 days including 2 resting days, leading to a total dose of 26 Gy in the µCT and 11 Gy in the RS225. The comparably low total dose was chosen to enable the detection of a potential improvement by adding the BaNPs. In the case of the RS225, the non-tumor area was also shielded and in contrast to the CT, the irradiation was only performed from above the tumor.

### Irradiation effects in cultured tumor cells

**Fig. 2:**
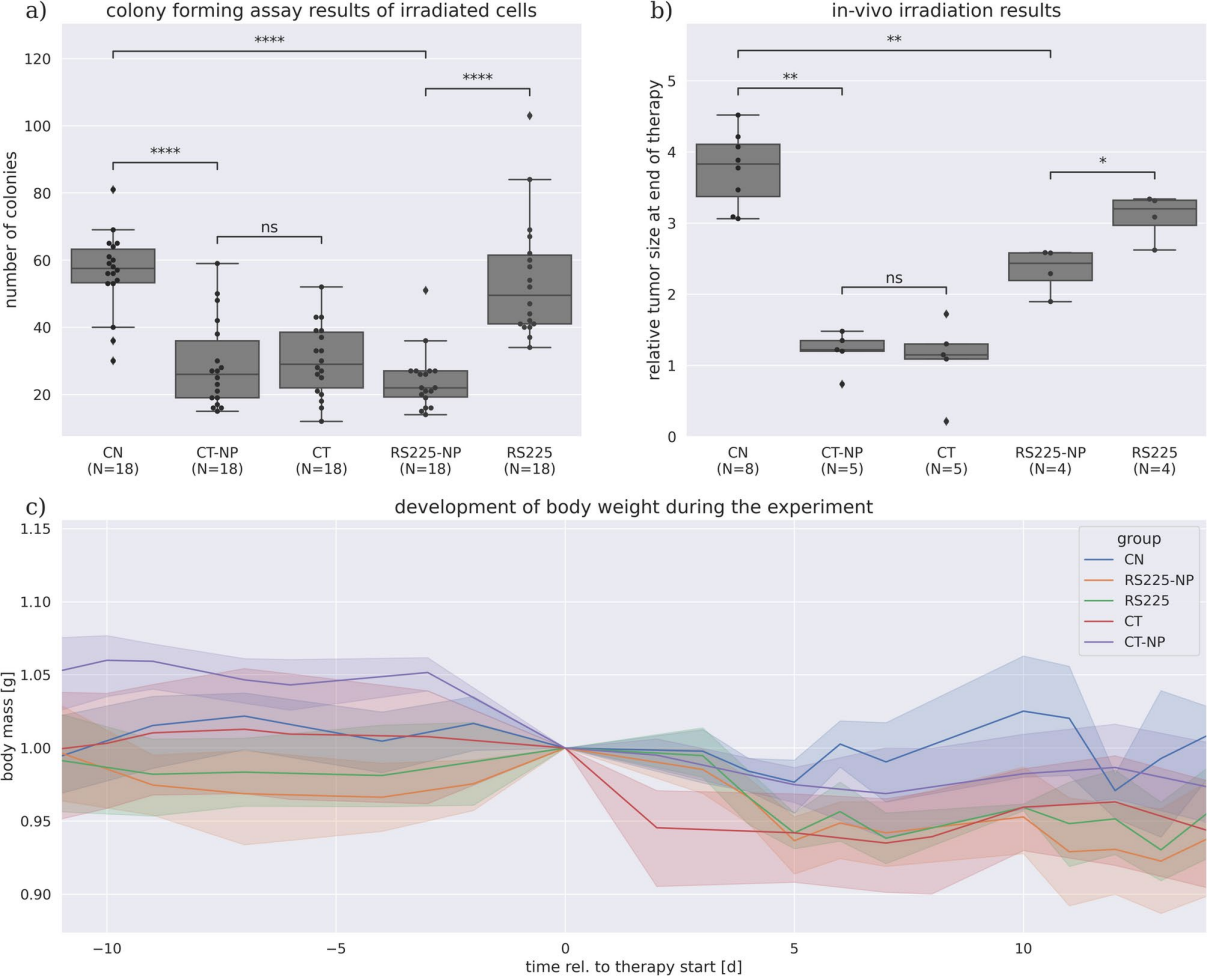
Effect of BaNPs in combination with CT-based or conventional RT. (**a**) shows the in-vitro results. CT irradiation with a 1.3 Gy exposure effectively reduces the colony forming ability of murine H8N8 breast tumor cells, but the BaNPs do not show an additional effect. 1.1 Gy exposure using the RS225 leads to a significant decrease of the colony forming ability of the tumor cells co-cultured with the BaNPs compared to irradiated cells only as well as to the controls. (**b**) shows the in-vivo results as relative tumor sizes (tumor size at the end of the experiment normalized to the size at the beginning of the treatment). A similar trend to the in-vitro results is found, where CT irradiation with a total dose of 26 Gy resulted in a strongly reduced tumor growth without an additional effect of the BaNPs. Irradiation with RS225 and a dose of 11 Gy resulted in a significant difference between tumors with and without injected BaNPs. c) development of the body weight in the individual experimental groups normalized to the start of the treatment (day 0). A moderate weight loss in all irradiated groups of maximally 5% was observed. (*ns* = not significant, * = *p*<0.05, ** = *p*<0.01, *** = *p*<1e-3, **** = *p*<1e-4).

We used a standard colony forming assay^21^ to analyze the irradiation effects on the breast tumor cells in-vitro. Figure 2a shows in comparison to non-irradiated cells (CN) that murine H8N8 breast tumor cells irradiated in the CT either with (CT-NP) or without BaNPs (CT) had a significantly reduced ability to form colonies. This proves, that the presence of BaNPs (CT-NP) in this setup did not have a significant additional impact on the reduction of cell growth (CT-NP vs. CT). In contrast, by using the RS225, the cells in combination with the BaNPs (RS225-NP) showed the same reduced ability to form colonies as the cells irradiated in the CT. The cells without BaNPs (RS225) seemed nearly unaffected by the radiation. Thus, BaNPs did enhance the effectiveness of the treatment at this condition, approximately to the same level found for the cells irradiated within the CT. Note that all cell irradiations were performed on cell pellets in 1.5 ml tubes after being co-cultured with the BaNPs for 24 h.

**Fig. 3:**
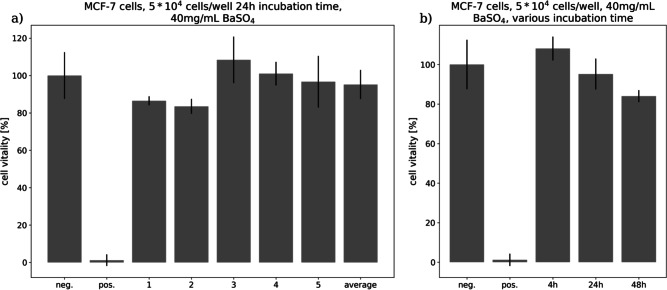
Cell vitality results after BaNP exposure. 5x10^4^ human MCF-7 breast cancer cells per well were exposed to 40 mg/ml BaNPs for 4, 24 or 48 h. BaNP treated cells did not show a significant difference in the cell vitality to non-treated controls (neg.).

To exclude potential toxicity of the BaNPs a colorimetric assay for assessing cell metabolic activity was performed. Figure 3a demonstrates that no toxicity was found for human MCF-7 breast cancer cells exposed to 40 mg/ml BaNPs for 24 h. The average of all analyzed wells showed a cell vitality of nearly 100% close to the negative control and far apart from the nearly 0% of the positive control. Figure 3b shows that the results were consistent at different time points (4, 24 and 48 h). All values were obtained from six replicates.

### Nanoparticle enhanced radiation therapy using a preclinical in-vivo CT system

Based on the in-vitro results we used the same settings for the in-vivo study. We implanted H8N8 cells in the mammary fat pad of WAP-T-NP8 mice, a reproducible model which leads to palpable tumors within 2 weeks and to volumes of about 500 mm^3^ within $\approx$30 days of tumor cell injection. The growth kinetics of the tumors are very similar among mice and tumor-bearing mice showed no significant weight change during disease progression^16^. We observed successful tumor development in 96% of the mice with an average tumor volume at therapy start of 314 mm^3^ and a relative standard deviation in the volumes of 34%. All mice reached this tumor volume at approximately the same time point after implantation (22 days) with a variation of about 2 days. To account for small differences in the tumor volume at treatment start, we calculated the relative volume change as shown in Fig. 2b by dividing the tumor size at the end of the experiment by the start volume. The untreated control tumors reached a relative tumor volume of approx. 3.7, so roughly 4 times the start volume within the 12-day experiment (Note that 3 mice were sacrificed earlier as the tumor size had already reached the termination criteria). The tumors treated in the CT with a total dose of 26 Gy showed a significant reduced relative tumor volume. However, no significant enhanced treatment effect was observed in the presence of the BaNPs, in agreement with the in-vitro results. The treatment with a total dose of 11 Gy in the RS225 was less effective. However, a significant reduction in relative tumor sizes was found when BaNPs were injected. Figure 2c shows the development of the body weight of the laboratory mice within the individual groups over the course of the experiment. The body weight was normalized to day 0, the day on which the irradiation was started (in case of the control group CN the same day was used). We found a moderate reduction in body weight in all groups apart from the untreated control group (CN), with a maximal relative weight loss in the range of 5%.

### Validation of particle distribution within the tumor

The kinetic and distribution of the BaNPs within the tumor seems to be of paramount importance for the treatment effect. Thus, we analyzed the distribution of the particles after *intra-tumoral* injection in the CT data sets. We found two different distribution patterns: (a) inhomogenous distribution within the tumor and (b) accumulation predominately in the capsule of the tumor. In both cases we did not find any decrease in the volume of the NP-positive regions within the tumors throughout the duration of the experiment. The volumes were calculated by simple threshold based segmentation in the CT scans of the groups treated with low photon energy RT in the CT. This information was not obtained for the groups treated in the RS225 irradiation device as no CT scans were performed.

**Fig. 4:**
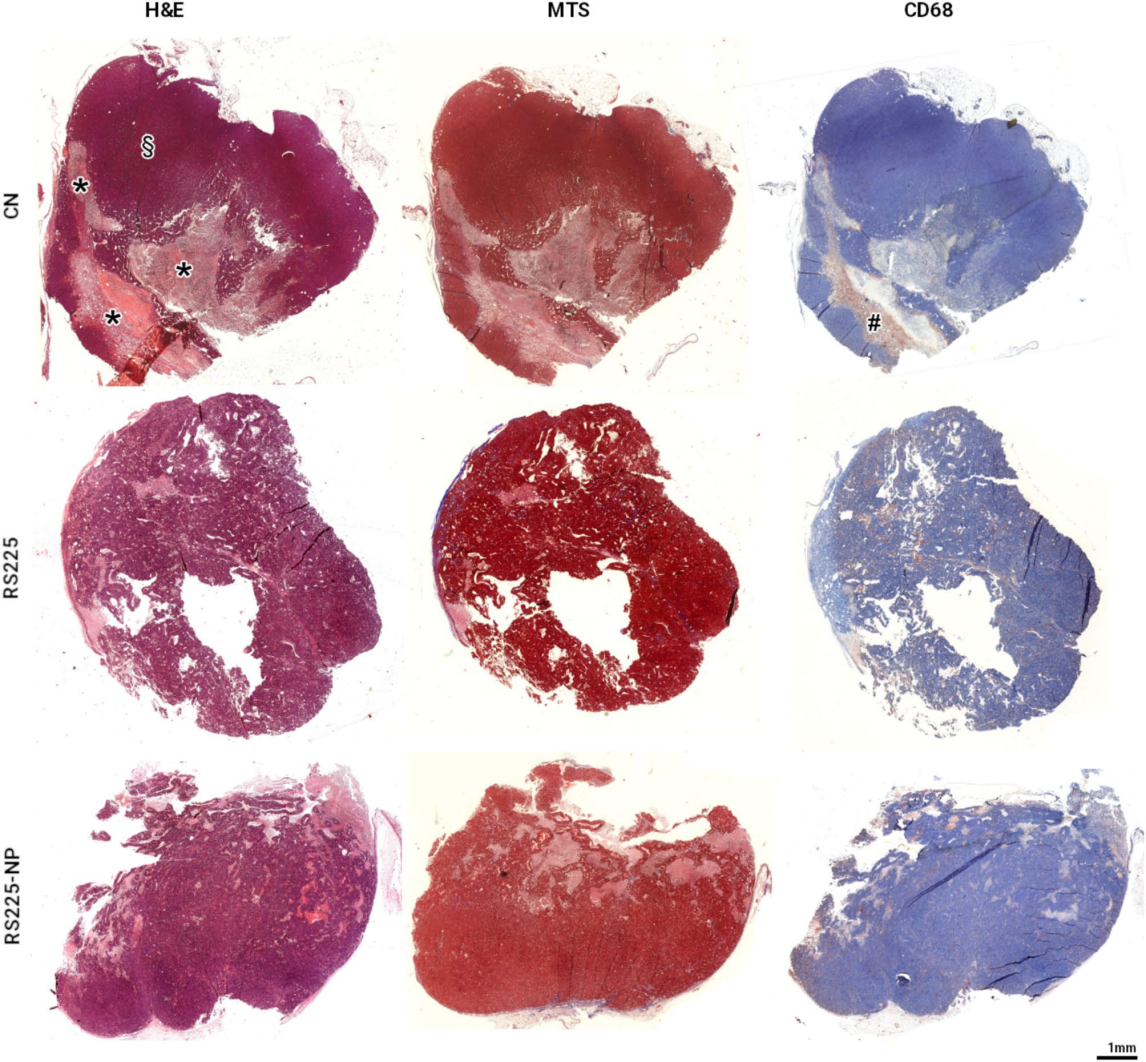
Histological appearance of the H8N8 breast tumors after conventional RT. Representative images of an untreated tumor (CN), a tumor that received RT (RS225) and a tumor that received RT and BaNPs (RS225-NP) are shown. H&E staining demonstrates large necrotic regions (asterisks) in tumor tissue of CN, while the tumor mass seems compact (§). RS225 and RS225-NP treated tumors appear more fractured and display small but distributed necrotic tumor regions. MTS staining confirms the absence of fibrosis in all specimens. CD68 staining shows a large mass of CD68 positive cells (predominantly macrophages) in the necrotic area in CN (#), while absent in the rest of the tumor. In RS225 and RS225-NP treated tumors, CD68 positive regions can be found distributed within the entire tumor. Note that the tumors were divided before paraffin embedding and might therefore appear incomplete.

**Fig. 5:**
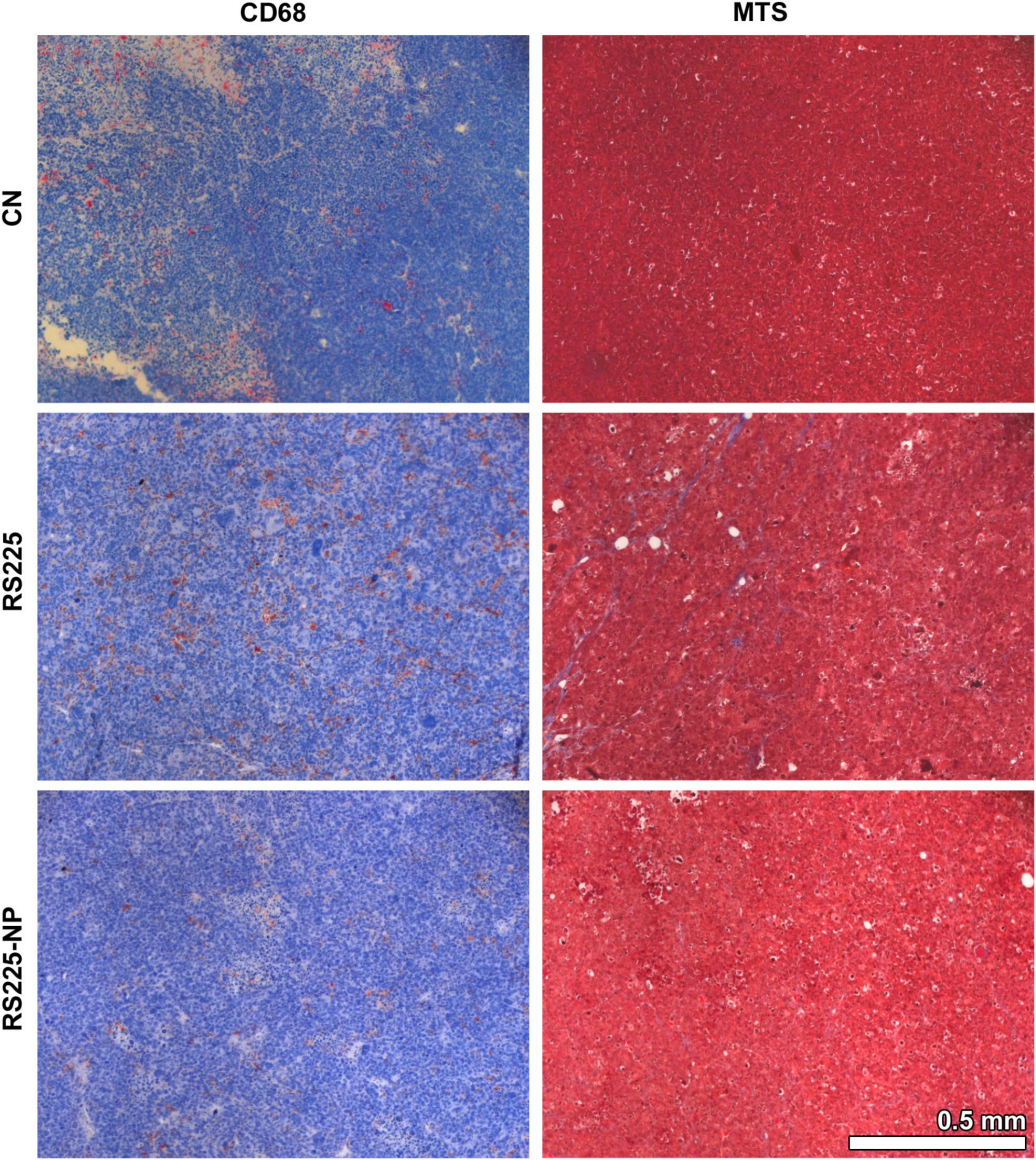
Detail views of representative areas from the same examples of a non-irradiated tumor (CN), a tumor irradiated with the RS225 device (RS225) and a tumor irradiated in the RS225 after injection of BaNPs (RS225-NP). Both the anti-CD68 antibody staining as well as the Masson Goldner Trichrome staining (MTS) revealed no major difference in the amount of macrophages (brown, CD68) or fiber content (blue, MTS).

To validate the in-vivo findings, the tumors were harvested and processed using the standard workflow for paraffin-embedded tissue analysis. Figure 4 shows that due to their small sizes, the BaNPs were not visible in classical H&E stained histology. The most striking difference between CN, RS225 and RS225-NP was the presence of larger necrotic areas and a more compact region of vital tumor mass in CN (Fig. 4). The detail views of the anti-CD68 antibody staining as well as the Masson Goldner Trichrome staining (MTS) shown in Fig. 5 validated no major differences in the macrophage concentration (CD68) or in the fiber content (MTS, blue) in all examples of the three different groups.

**Fig. 6:**
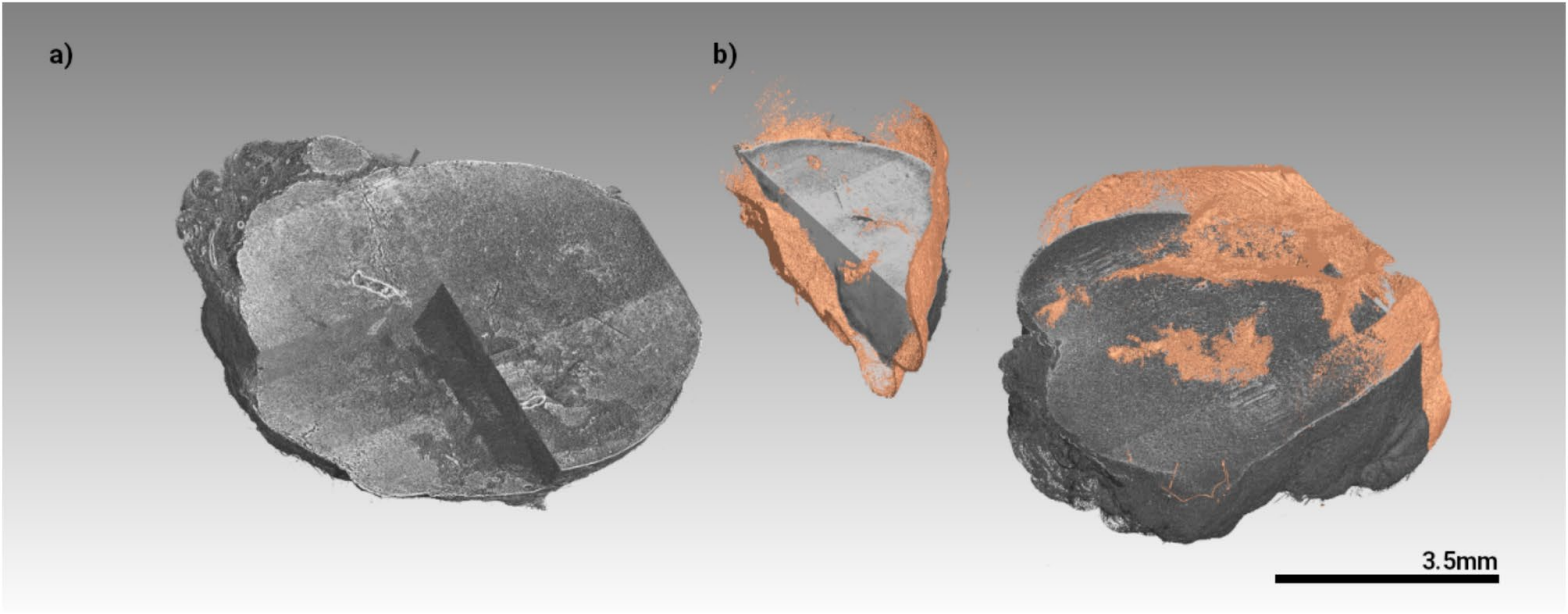
BaNP distribution in the tumors analysed by synchrotron based phase contrast µCT. Representative images of (**a**) a breast tumor that received RT (RS225) and (**b**) a breast tumor that received RT and BaNPs (RS225-NP), showing the BaNPs distribution in the tumor capsule (golden). The tumor tissue is displayed clipped to a smaller size than the 3D rendering of the BaNP distribution to facilitate better visualization. Please note that the tumor was cut in the middle before paraffin embedding. Thus (**b**) shows two pieces of the same tumor.

Prior to sectioning, the paraffin blocks were scanned at the beamline SYnchrotron Radiation for MEdical Physics (SYRMEP) of the Italian synchrotron using the white beam phase contrast CT setup with a resolution of 2 µm as described by Dullin et al.^22^. In contrast to classical histology, phase contrast CT allowed the visualization of the BaNPs as well as the soft tissue of the tumor in 3D (Fig. 6). This ex-vivo analysis confirms the distribution pattern observed in the in-vivo CT studies by showing the majority of BaNPs in the periphery of the tumor. Exemplary, the lung and liver of two tumor bearing mice - one injected with BaNPs, one without - were imaged as well (Suppl. Fig. S1). In the lung we found small dense structures in both the BaNPs injected mouse as well as in the control. Despite the strong contrast in the BaNP injected tumor, we also observed dense structures in the control tumor, which most likely represent hemorrhage. Regarding the liver, more dense structures were found in the liver of the BaNP injected mouse, suggesting that part of the particles were transported from the tumor site and were taken up by the liver. However, the number of BaNP-associated dense regions in the liver and lung was only a fraction of that found in the primary tumor, suggesting that most of the particles remained at the tumor site during the entire treatment procedure.

## Discussion

Here we demonstrated that *intra-tumoral* injected barium-based NPs can be used to boost the efficacy of x-ray based external radiation therapy, when combined with the appropriate photon energy. Despite the fact that the absorption maximum of barium at 37.4 keV is well suited for CT imaging, by using a CT as an irradiation device, we did not observe an improvement of the therapeutic effect in the presence of BaNPs. By contrast, at an average photon energy of 90 keV as used in the RS225 irradiation device, we achieved an approx. 2-fold increase in the anti-tumoral effect of RT in-vitro. In-vivo irradiation of mouse breast tumors without BaNPs resulted in an approx. 17% reduction in the tumor growth rate, which increased to a 38% reduction in combination with BaNPs.

Interestingly, even the cells irradiated without BaNPs showed a strongly reduced ability to form colonies after the irradiation within the CT but not in the group irradiated in the RS225. This shows that the low-energy radiation of 40 keV does more damage to the cells than the radiation at 90 keV. The results obtained in the presence of the BaNPs at 90 keV suggest that the efficacy of the RT can be raised, while at the same time reducing the effects in areas without BaNPs. While we found that the amount of the injected BaNPs within the tumor did not diminish during the experiment, the distribution was fairly inhomogeneous. Thus, we believe that the success of the treatment in the RS225 system is based on the fact that the emitted photo-electrons have a higher kinetic energy and can penetrate up to 9 cell layers as opposed to the estimated 0.5 layers for the irradiation in the CT (if we assume a typical cell has a diameter of 10 µm). The fact that the BaNPs did not homogeneously distribute within the tumor is most likely a consequence of the local *intra-tumoral* injection in three fractions. First tests with functionalized BaNPs and their systemic application did not result in a sufficient tumor uptake and are therefore not shown in this manuscript. Thus, the route of application needs to be reconsidered and optimized. Furthermore, one of the main advantages of irradiation therapy is its unspecific nature, which allows its application for virtually any tumor entity. A combination with particles that need functionalization to reach the tumor site would diminish this aspect. However, at this point, the need of a local administration limits or prevents a translation to patient application.

We did find a slight increase of dense material within the liver of animals injected with BaNPs. Therefore, BaNPs must have been transported to this location from the tumor site. However, the liver was not part of the irradiated area of the mouse. Thus, a low uptake within the liver without activation can be tolerated in contrast to therapeutic particles.

Low energy radiation therapy in combination with contrast enhancers has already been proposed by several groups as for instance listed in the review by Bergs et al.^4^. Moreover, NPs have been used as radio-enhancers before, but are typically based on gold^23^, gadolinium^24^ or other high order elements such as hafnium^25^, bismuth^26^ etc. The k-shell electron of barium, that we used in our experiments, has a lower binding energy than in the previously used elements and will therefore receive a larger kinetic energy once emitted. We believe that this is a key factor in a setting such as ours, in which a homogeneous distribution of the NPs within the tumor cannot be achieved. Even at a low total dose of only 11 Gy we saw a two-fold reduction in the tumor growth rate when RT was combined with the BaNPs compared to irradiation alone. Since barium is ideally suited for in-vivo CT imaging and is therefore routinely applied as an oral contrast agent in the clinics, our BaNPs are not only enhancing the efficacy of RT, they also boost the tumor visibility in CT. The disadvantage of low energy RT in a higher keV range is the increase dose deposition not only in the tumor region but also in the healthy tissue above and within the skin. Therefore, small animal irradiation experiments commonly using 225 kV are critically discussed^27^. For a potential translation of our approach into patients the low photon energy to effectively use the BaNPs will most likely limit the application to surface-near tumor entities such as breast cancer. This disadvantage might be mediated by the enhancing properties of our BaNPs, which should allow working with an overall lower radiation dose.

In recent years, approaches to combine RT with chemotherapy and/or immunotherapy have been proposed. Denkova et al.^28^ reported the use of different strategies for the combination of RT and immuno- /chemotherapy. Radio-enhancer like our BaNPs might be ideally suited as a core compound for such an approach as the strong release of photo-electrons could be used to trigger a cascade of effects such as local release of chemotherapeutic drugs from combination NPs, an approach which is currently under evaluation.

The difference in tumor morphology observed by histology, in particular the larger necrotic areas in the untreated tumors, may be attributed to the faster tumor growth in this group. Thus, the absence of large necrotic areas in the treated specimens is most likely not a direct effect of RT but a consequence of the slower growth rate. However, the treated tumors with and without BaNPs showed local ruptured structures, which we believe were caused by the irradiation.

The ability of the photo-electrons generated in the irradiation device to reach tumor cells in a larger proximity of the BaNPs seems to outweigh the fact that the probability for x-ray absorption within the BaNPs drops to only 9% of the value at 40 keV. So far we only tested this one photon energy to limit the amount of animals. However, there could well be a sweet-spot between kinetic energy of the photo-electrons and probability of x-ray absorption, which could most likely further raise the effectiveness of the BaNPs.

A large variety of irradiation dose protocols for mouse tumor models have been reported as for instance summarized by Arnold et al.^29^. Our used total doses of 26 Gy or 11 Gy respectively seem well in line with the reported values. The body weight development of the treated mice during the experiment showing a maximum decrease of about 5% is a further indicator that the chosen dose protocol was well tolerated by the animals. However, as for the photon-energy a rigorous optimization might further improve the therapy outcome.

## Conclusion

Low energy RT was successfully applied in a breast cancer mouse model resulting in a reduction in tumor growth. The anti-tumoral efficacy of this treatment was increased by barium-based NPs. The discrepancy between the strong effects in cell assays and the limited effects in-vivo seems to be attributed to the limited range of the RT effect in combination with an inhomogeneous distribution of the BaNPs in the tumor. Thus, BaNP-enhanced RT may be a promising tool to lower the side effects of external beam RT on normal cells if a better BaNP distribution within the tumor can be achieved. Standard CT systems can provide sufficient doses for RT but, in combination with barium-based NPs, they are not able to generate photo-electrons with sufficient kinetic energy to deeply penetrate the tumor.

## Methods

### Irradiation devices

For irradiation two devices were used: an in-vivo µCT (QuantumFX, Perkin Elmer) and a cabinet irradiator for preclinical research (RS225, XStrahl). The µCT was used with the following parameters: tube voltage 90 kV, tube current 200 µA, field-of-view 20x20 mm², total irradiation time 2 min resulting in an x-ray dose of approximately 1.3 Gy and an average beam energy of 40 keV. The RS225 was utilized with 200 kV resulting in an average beam energy of 90 keV. Due to technical problems, the dose rate for the in-vivo experiment within the RS225 was 0.55 Gy/min instead of the 1.1 Gy/min used for the cell irradiation. The dose rate in the µCT was 0.65 Gy/min. In both cases for the in-vivo and cell irradiation experiments, dose rates were retrospectively validated by TLD measurements.

### Nanoparticles

**Fig. 7:**
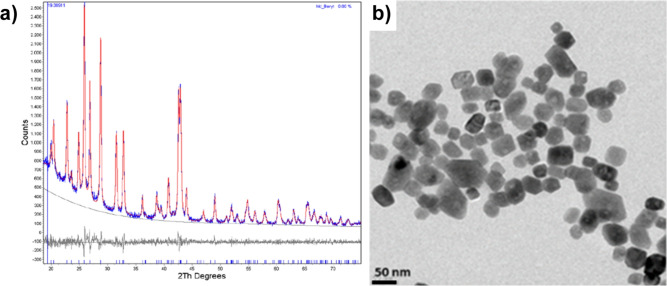
Barium nanoparticle design. (**a**) X-ray diffraction pattern (blue) and calculated pattern based on baryte using Rietveld-Analysis (red). The gray curve represents the remaining difference between the intensity of the blue and red curve. No other crystalline phases were detected. (**b**) Typical TEM micrograph of the obtained BaNPs.

Barium sulphate (BaSO_4_)-based nanoparticles (BaNPs) with a hydrodynamic diameter of 120 nm were produced by chemical precipitation at ambient conditions. Barium sulphate (BaSO_4_) nanoparticles (BaNPs) were produced by chemical precipitation in aqueous solution at ambient conditions according to: ${Ba^{2+}+SO_4^{2-}{\rightarrow }{BaSO_4}{\downarrow }}$. The obtained white suspension was homogenized using an ultraturrax dispersing device. After a first purification step, a biocompatible polymer for steric stabilization was added. The suspension was dialyzed against water to remove excess ions and finally concentrated to a mass concentration of 200 mg/mL BaSO_4_. (All other experimental details cannot be disclosed as they are IP of nanoPET Pharma). The medium core diameter of the obtained highly pure and crystalline nanoparticles (Fig. 7a) was d = 43 nm determined by transmission electron microscopy (Fig. 7b) and the hydrodynamic diameter was d_h_ = 120 nm determined by dynamic light scattering method. The highly stable colloidal suspension was steam sterilized and formulated under physiological conditions for in-vitro/in-vivo use.

### Cell culture

Murine H8N8 breast tumor cells were cultured in a T-75 cell culture flask in high Glucose (4.5 g/L d-Glucose) Dulbecco’s modified Eagle’s medium (DMEM, Thermo Fisher Scientific Corp., Waltham, MA) containing 10% fetal calf serum (FCS, Thermo Fisher Scientific Corp., Waltham, MA) at 37 °C in a humidified atmosphere with 5% CO_2_. When confluence was reached, the cells were washed with 10 mL phosphate-buffered saline (PBS). The adhesive cells were removed from the flask by adding 5 mL trypsin/EDTA. Trypsination was stopped after 5 min by adding 10 ml cell culture medium containing FCS. The cells were co-cultured for 24 h with 40 mg/ml BaNPs. The cells were removed from the well-plates and centrifuged for 2 min at 1200 rpm. The cell pellet was resuspended in 4 ml PBS and distributed into four 1.5 ml E-cups. Before irradiation the cells were gently centrifuged again, and PBS was removed with a pipette. Subsequently, the cells were irradiated either in the µCT using an x-ray dose of approximately 1.3 Gy or in the RS225 with 1.1 Gy. After irradiation, the cells were resuspended in cell culture medium and counted using a Neubauer chamber. 1000 cells per well were seeded in 6-well plates. One plate was used per treatment group per experiment.

A cell proliferation assay (MTT-assay) was performed using the human MCF-7 breast cancer cell line to assess cytotoxicity of the BaNPs. The experiments (N = 6 replicates) were conducted at 4 h, 24 h and 48 h incubation time and with a final BaNP concentration of 40 mg/ml. The results were compared against a positive (100% dead cells) and negative control (100% vital cells) as described in Ramos et al.^30^.

### Colony forming assay

The cells were cultured in media for 7–10 days at 37 °C and 5% CO_2_ until the colonies were large enough for imaging but did not start to merge. The cell culture medium was removed, and each well was washed with 2 ml PBS. The cells were fixed by adding 1 ml/well of 70 % EtOH for 15 min. The plates were left open to dry on the workbench. To stain the cells, 1 ml/well of crystal violet staining solution was added and incubated for 5 min. The staining solution was removed and the wells were rinsed gently with demineralized water followed by a bluing step for 5 min under a light tap water flow. The cell culture plates were left open in the workbench until they were completely dry. A flatbed scanner (Epson Perfection V800) was used in transillumination mode to image the wells at 6400 dpi. A custom-made python based software pipeline was then applied to automatically count and analyze the colonies.

### Tumor model, experimental design and irradiation

1x10^6^ H8N8 tumor cells suspended in 20 µl PBS were orthotopically implanted into the right abdominal mammary gland of ketamine/xylazine anesthetized (2.5–3 µl/g body weight) syngeneic female WAP-T-NP8 mice (N = 18) as described in Pantelaiou-Prokaki et al.^16^. Transplanted animals were checked and weighed three times a week for the entire duration of the experiments. Irradiation treatment was started when a tumor volume of 300 mm^2^ was reached. Tumor volumes were measured using a caliper and estimating the volume with the formula $$v=(A*B*C)/2$$, with A, B and C being the dimensions in x, y and z direction, respectively. The undiluted BaNPs solution (300 mg/ml) was injected directly into the tumor 24 h prior to the 1^st^ irradiation. To attempt a homogeneous BaNPs distribution in the tumor, 3 × 10 µl of BaNPs solution were injected with a 10 µl Hamilton glass syringe at three different sites of the tumor. One group (N = 10) was irradiated with the µCT using a total dose of 26 Gy (5 days 2.6 Gy per day, 2 resting days, 5 days 2.6 Gy per day). Five mice of this group were injected with BaNPs (CT-NP, N = 5) and five did not receive particles (CT, N = 5). Another group of 8 mice (N = 8) was irradiated with the RS225 device using a total dose of 11 Gy (5 days 1.1 Gy per day, 2 resting days, 5 days 1.1 Gy per day). Four mice of these mice were injected with BaNPs (RS225-NP, N = 4), and four did not receive nanoparticles (RS225, N = 4). 24 h after the last treatment the mice were sacrificed using CO_2_ in combination with cervical dislocation. The tumors and organs were harvested, formalin-fixed for 24 h, chemically dried using a standard ascending ethanol series and then embedded in paraffin.

### Phase contrast µCT and histology

The entire blocks were scanned at the SYRMEP beamline of the Italian synchrotron “Elettra” using the following settings: white beam mode, 0.5 mm silica filter, sample-to-detector distance 15 cm, resolution 2 µm. Up to 3 overlapping scans were performed to achieve an overall field-of-view of about 8x8x10.5 mm. Single distance phase retrieval was applied with a delta-to-beta ratio of 100 prior to reconstruction using filtered back propagation, both implemented in the SyrmepTomoProject (STP)^31^. A custom made python script was used to stitch the data together. Following phase contrast CT, 2 µm paraffin section were cut using a microtome, deparaffinized and stained with haematoxylin and eosin (H&E), Masson Goldner Trichrome (MTS) and an anti-CD68 antibody (CD68) as described in^14^. Stained slices were then imaged with an Axiovert 200 inverted microscope (Zeiss) and stitched to images showing the entire cross-section of the tumor.

### Ethical statement

All animal in-vivo procedures were performed in compliance with the guidelines of the European Directive (2010/63/EU) and the German animal ethics regulations and were approved by the local ethics office (Niedersaechsisches Landesamt für Verbraucherschutz und Lebensmittelsicherheit, LAVES, ethics approval AZ33.9-42502-04-18/3022). Furthermore, the study was carried out in compliance with the ARRIVE guidelines (https://arriveguidelines.org).

### Software and statistics

All statistical analysis and plotting of the results was done in Python with matplotlib (https://matplotlib.org), statannot (https://pypi.org/project/statannot), seaborn(https://seaborn.pydata.org) and pandas (https://pandas.pydata.org). We used a “Mann-Whitney” test to account for the comparably low number of samples. A* p*-value of less than 0.05 was considered statistically significant.* P*-values are labelled in the graphs as follows *p* < 1e-4=“****”, *p* < 1e-3=“***”, *p* < 0.01=“**” and *p* < 0.05=“*”. Segmentation and rendering of the cancer region in Fig. 5 was done in Scry 6.0, a custom-made software by the author. Displaying the large phase contrast CT data sets of the paraffin-embedded tumors and selecting a virtual cut corresponding to the histological section was done in VGStudioMax 3.4.0 (Volume Graphics, Heidelberg, Deutschland). Preprocessing of the microscope images was done in Fiji^32^.

## Supplementary Information

Supplementary Information.

## Data Availability

Due to the fact that especially the data sets of the phase contrast CT acquisitions are very large, they cannot be provided in an online repository. Thus, the data is available under reasonable request from C.Dullin (mailto:christian.dullin@protonmail.com).
