# Supplementary material for: Barium nanoparticles enhance efficacy of external beam radiation therapy in a preclinical basal-like mammary cancer mouse model
Source: Sci Rep. 2025 May 30;15:19090. doi: 10.1038/s41598-025-02560-4 (PMC12125400; doi:10.1038/s41598-025-02560-4)
Supplement: Supplementary file 1 — Supplementary Information. [file 41598_2025_2560_MOESM1_ESM.pdf]

Barium nanoparticles enhance efficacy of external beam radiation therapy in a preclinical basal-like mammary cancer mouse model

Jonas Albers[1,2], Andrea Markus[3], Angelika Svetlove[2,3], Alexander Kraupner[4], Andreas Briel[4], Frauke Alves[1,3,5], and Christian Dullin[1,3,6,7]

Supplemental Materials

Figure Legend – Supplemental Figure S1:

Barium nanoparticle distribution. Depicted are slices from phase contrast microCT scans of lung, liver and tumor of one mouse injected with NPs (CT-NP) and one without (CT). Some dense structures can also be seen in the organs of the control mouse (most likely blood cells). a) and b) show only minimal dense structures in the lung in both cases. The liver of the BaNP injected mouse (c) shows more contrast than that of the non-injected mouse (d), suggesting that part of the NPs traveled from the tumor and accumulated in the liver. e) and f) A strong contrast is clearly seen in the tumor injected with BaNPs, but some contrast was also detected in the tumor of the control mouse (f) suggesting a signal based on the iron content of a hemorrhage. Top panels show zoomed in images of regions of interest marked in the lower panels.

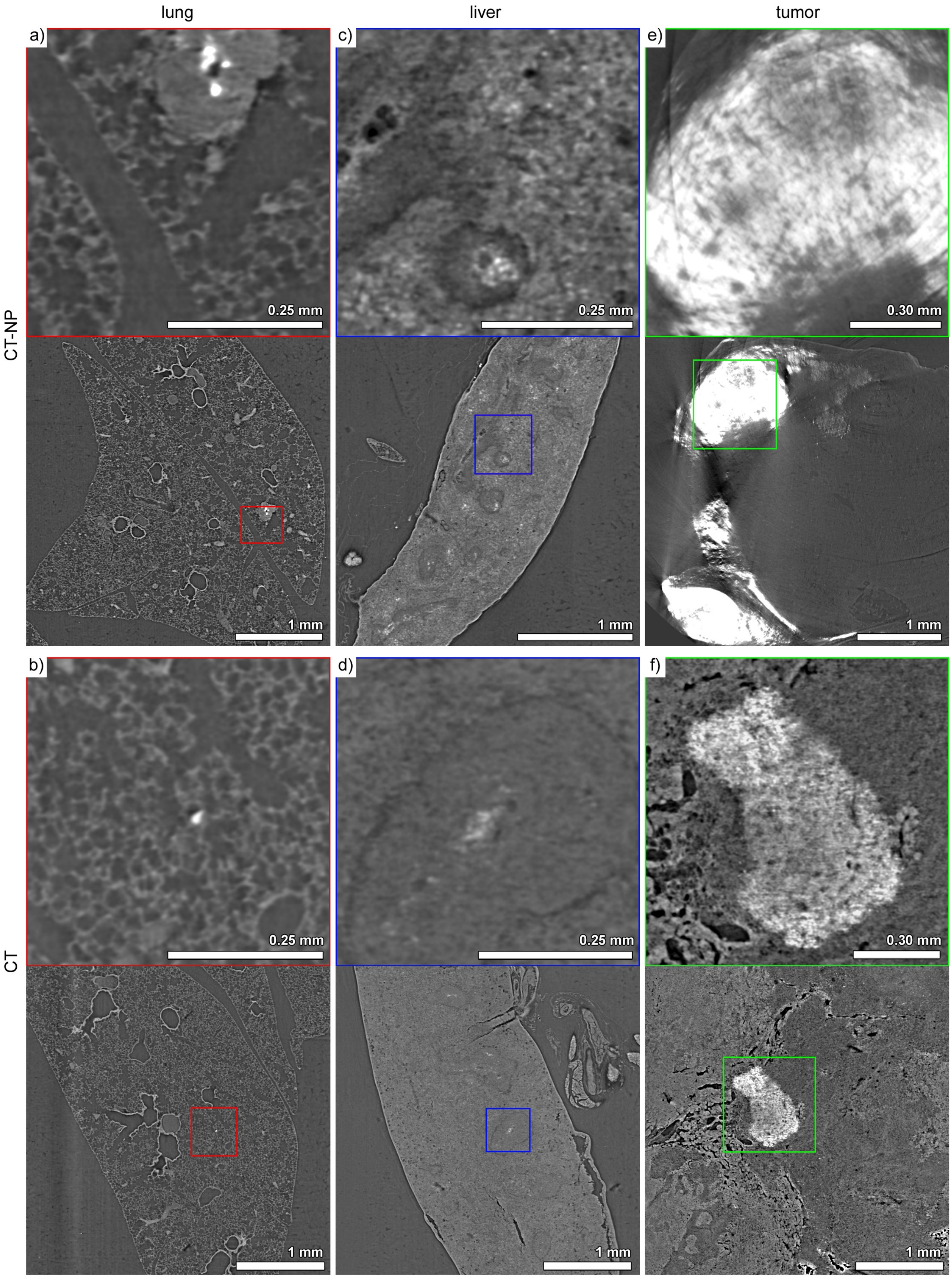

Figure 1: Suppl. Figure S1
